# Resection of Esophageal Carcinosarcoma with Residual Sarcoma Post-Chemoradiotherapy: A Case Report

**DOI:** 10.70352/scrj.cr.25-0618

**Published:** 2025-12-16

**Authors:** Ryo Shibayama, Yutaka Takazawa, Kentoku Fujisawa, Yusuke Ogawa, Hayato Shimoyama, Shusuke Haruta, Masaki Ueno

**Affiliations:** 1 Department of Gastroenterological Surgery, Toranomon Hospital, Tokyo, Japan; 2 Department of Pathology, Toranomon Hospital, Tokyo, Japan

**Keywords:** carcinosarcoma, esophageal cancer, chemoradiotherapy

## Abstract

**INTRODUCTION:**

Esophageal carcinosarcoma is a rare malignancy with both carcinomatous and sarcomatous components. Radical resection is the treatment of choice; however, there is disagreement about whether to perform perioperative chemotherapy or chemoradiotherapy. We report a case of radical resection of esophageal cancer with a residual sarcomatous component in the operative specimen following chemoradiotherapy for suspected esophageal carcinosarcoma.

**CASE PRESENTATION:**

A 60-year-old man with a chief complaint of dysphagia who underwent esophagogastroduodenoscopy was diagnosed with esophageal squamous cell carcinoma and treated with definitive chemoradiotherapy. Nine months later, esophagogastroduodenoscopy showed an elevated lesion, and biopsy confirmed a pathological diagnosis of spindle cell sarcoma. We hypothesized that the final course of chemoradiotherapy therapy would eliminate the esophageal squamous cell carcinoma but allow the sarcoma to progress quickly. No metastasis was detected, and the tumor was suspected to be highly malignant; therefore, esophagectomy was performed as part of a 3-field lymphadenectomy. The histopathological diagnosis was esophageal cancer with a residual sarcomatous component in the operative material. Postoperative examination of the original esophageal squamous cell carcinoma revealed poorly differentiated keratinocytes, indicating the presence of mixed sarcomatous components. We concluded that the carcinomatous component disappeared following chemoradiotherapy for esophageal carcinosarcoma, whereas the sarcomatous component remained.

**CONCLUSIONS:**

This case represents an esophageal carcinosarcoma in which the carcinomatous component completely regressed after definitive chemoradiotherapy, whereas the sarcomatous component persisted and required surgical resection. This finding indicates a differential therapeutic response between the two components of carcinosarcoma and provides important insight into the management of this rare tumor.

## Abbreviations

dCRT
: definitive chemoradiotherapy
EGD
: esophagogastroduodenoscopy

## INTRODUCTION

Esophageal carcinosarcoma is a rare malignancy of the esophagus composed of both carcinomatous and sarcomatous components, accounting for 0.6% of all esophageal malignancies.^1)^ Radical resection is the preferred treatment for this condition. There is no consensus regarding the use of perioperative chemotherapy or chemoradiotherapy; however, some studies have reported their usefulness.^2,3)^ Moreover, one report has described varying degrees of therapeutic effects of radiation chemotherapy for carcinosarcoma on the carcinomatous and sarcomatous components.^4)^ We report a case of radical resection of esophageal cancer with a residual sarcomatous component identified in the operative specimen following chemoradiotherapy for suspected esophageal carcinosarcoma.

## CASE PRESENTATION

**Fig. 1:**
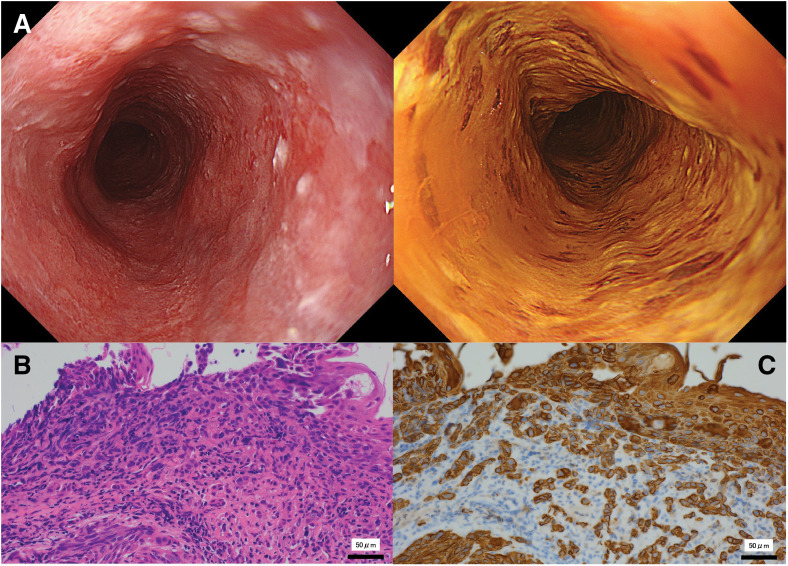
Esophagogastroduodenoscopy and biopsy findings. Esophagogastroduodenoscopy revealed a type 0-IIb flat lesion extending from the esophageal inlet to the lower thoracic esophagus (18–39 cm from the incisors), and squamous cell carcinoma was detected on biopsy (**A**). H&E staining shows carcinoma cells proliferating within the mucosal epithelium and infiltrating the stroma, forming small nests (**B**). Immunostaining for cytokeratin (AE1/AE3) shows a cord-like and single-cell infiltrative pattern with spindle-shaped tumor cells, suggesting early sarcomatous differentiation (**C**). H&E, hematoxylin and eosin

A 60-year-old man with a chief complaint of dysphagia underwent EGD, performed by his previous physician, and was diagnosed with esophageal squamous cell carcinoma. He had no significant medical history, except for a 25-year history of smoking 20 cigarettes per day and occasional alcohol consumption. EGD revealed a type 0-IIb flat lesion extending from the esophageal inlet to the lower thoracic esophagus (18–39 cm from the incisors), and biopsy confirmed squamous cell carcinoma with poorly differentiated features (**Fig. 1**). Although an endoscopic image is not shown, preoperative endoscopy confirmed that the proximal esophagus was free of tumor involvement, which justified performing subtotal instead of total resection. CT showed no lymph node or distant metastases, and the tumor was classified as cT1N0M0 stage I according to the TNM classification of the UICC 8th edition. Considering that the lesion was long on the oral side, laryngectomy would have been required if resection had been chosen; therefore, a dCRT was preferred. The irradiation dose was 60 Gy/30 Fr. The chemotherapy regimen consisted of cisplatin (70 mg/m^2^ on day 1) and 5-fluorouracil (700 mg/m^2^/day, days 1–4).

**Fig. 2:**
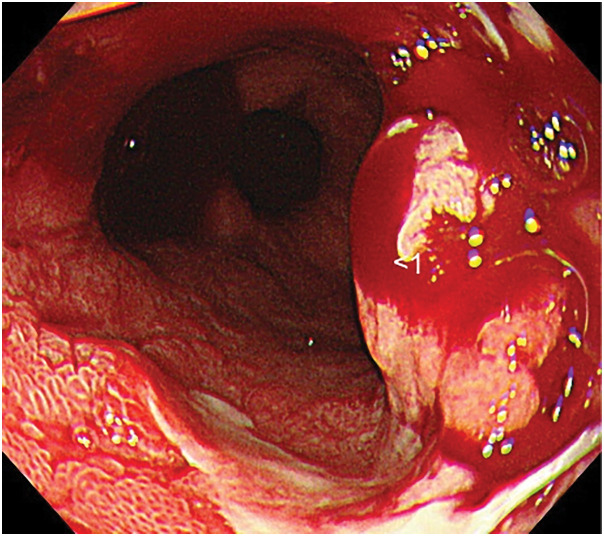
Esophagogastroduodenoscopy performed 9 months after dCRT. This figure reveals an elevated lesion on the anorectal side of the ulcer. A step biopsy was performed to confirm tumor negativity on the oral side, and a small number of spindle cells were identified within the granulation tissue located 27–38 cm from the incisors. dCRT, definitive chemoradiotherapy

**Fig. 3:**
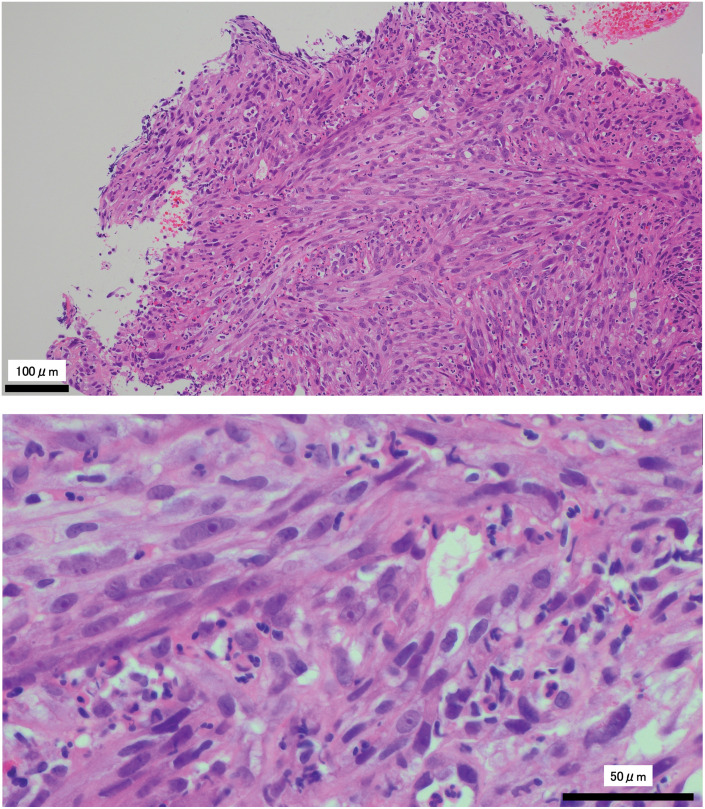
Spindle cells are observed to diffusely proliferate in a fascicular arrangement. H&E staining reveals atypical spindle cells characterized by swollen nuclei and prominent nucleoli. H&E, hematoxylin and eosin

EGD performed at 1, 3, and 5 months after completion of dCRT revealed circumferential post-CRT ulceration, which was interpreted as treatment-induced scarring rather than residual disease. Furthermore, EGD performed 9 months after dCRT showed an elevated lesion on the anorectal side of the ulcer (**Fig. 2**), and the biopsy confirmed spindle cell sarcoma (**Fig. 3**). The immunohistochemical findings were as follows: CK AE1/AE3 (−), CK CAM5.2 (−), p63 (−), Ki-67 LI 50%, desmin (−), S100 (−), α-SMA (focally +), MSA (focally +), KIT (−), DOG1 (−), and CD34 (−). A step biopsy was performed to confirm the absence of tumor spread on the oral side, and a small number of spindle cells were identified within the granulation tissue 27–38 cm from the incisors. We hypothesized that the presence of esophageal squamous cell carcinoma at 18–39 cm from the incisors suggested a possible complete response to dCRT. However, a new radiation-induced esophageal spindle cell sarcoma may have developed within a short interval, or an originally existing sarcoma may have manifested 27–38 cm from the incisors.

**Fig. 4:**
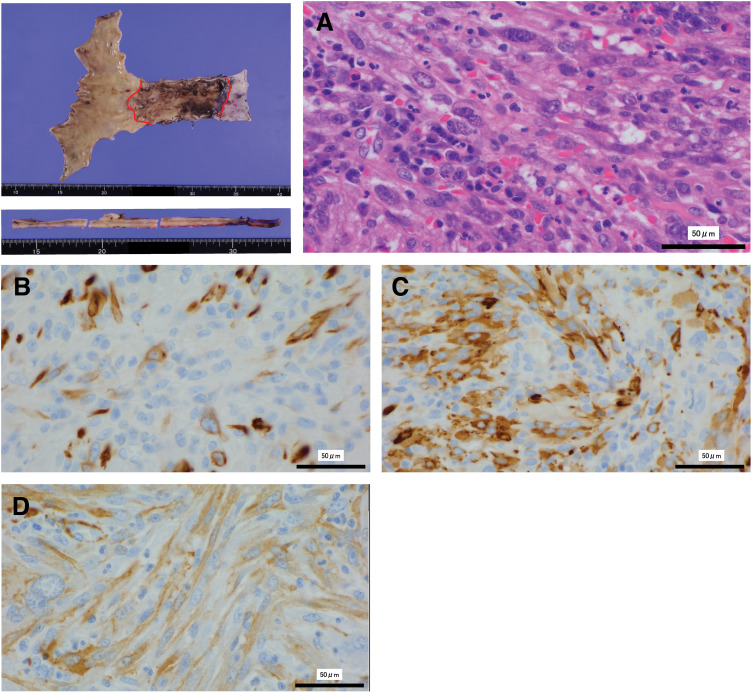
H&E stain, ×40. Atypical spindle cells, interspersed with lymphocytes, exhibit infiltrative and diffuse growth (**A**). Desmin, ×40. A few sarcoma cells are positive for desmin on immunostaining (**B**). h-Caldesmon, ×40. Most sarcoma cells are positive for h-caldesmon (**C**). α-SMA, ×40. Most sarcoma cells are also positive for α-SMA (**D**). H&E, hematoxylin and eosin

CT showed no evidence of metastasis, and the tumor was expected to be highly malignant. Therefore, an esophagectomy was performed as part of a 3-field lymphadenectomy. The operation lasted 495 min, with an estimated blood loss of 15 mL. No major postoperative complications occurred. Pathological findings showed spindle cell sarcoma with a leiomyosarcomatous phenotype in the elevated lesions of the operative specimen (**Fig. 4**). Sarcoma cells infiltrated the mucosal layer with erosive lesions on the oral side. The immunohistochemical findings of the sarcoma were as follows: desmin (focally +), α-SMA (focally +), HHF35 (focally and weakly +), caldesmon (focally +), CK AE1/AE3 (−), CK CAM5.2 (−), ERG (−), CD34 (−), CD31 (−), factor VIII (−), and S-100 (−). No lymph node metastasis was detected. The patient was under outpatient follow-up for 1 year postoperatively and survived without recurrence.

## DISCUSSION

In the present case, histological examination after chemoradiotherapy revealed the complete disappearance of the carcinomatous component and persistence of a sarcomatous component. Three possible mechanisms were considered to explain this finding:

1) the development of a radiation-induced esophageal leiomyosarcoma after chemoradiotherapy for squamous cell carcinoma;
2) pre-existing esophageal carcinosarcoma in which the carcinomatous component responded completely to chemoradiotherapy while the sarcomatous component persisted; and
3) the coincidental occurrence of esophageal squamous cell carcinoma and esophageal leiomyosarcoma as independent, heterochronous tumors.

Among these possibilities, the second hypothesis appears most plausible. The pretreatment biopsy (**Fig. 1C**) already showed spindle-shaped tumor cells suggestive of early sarcomatous differentiation, indicating that this tumor was a carcinosarcoma from the outset. Therefore, this case demonstrates that chemoradiotherapy can achieve a complete response in the carcinomatous element of esophageal carcinosarcoma while leaving the sarcomatous element viable.

Esophageal leiomyosarcoma is a rare tumor, accounting for less than 0.5% of all esophageal malignancies.^5)^ Approximately 60% of esophageal leiomyosarcomas present as polyp-like growths protruding into the esophageal lumen, while the remaining 40% manifest in other forms, all appearing as mass lesions.^6)^ Resection is the most important treatment for esophageal leiomyosarcoma; however, chemotherapy and radiation therapy have limited efficacy.^6)^

Esophageal carcinosarcoma accounts for 0.27%–2.8% of esophageal malignancies.^7–9)^ They are generally characterized by faster growth^10)^ and polyp-like development,^11)^ in contrast to esophageal cancer in general. Endoscopic biopsy may detect only epithelial components in 2/3rd of cases, often leading to a diagnosis of squamous cell carcinoma.^12)^ These tumors have large polyps with associated symptoms that appear early and show an outward proliferative growth pattern toward the lumen. More than 80% of cases have a high degree of malignancy, although the tumor’s primary location is limited to the submucosa or muscularis propria.^13)^

Immunohistochemical testing is important for the diagnosis of stromal tumors. Vimentin is used as an indicator of mesenchymal differentiation; α-SMA and desmin for muscle differentiation; and S-100 protein for nerve and cartilage differentiation.^14)^ Although molecular analysis and vimentin immunostaining, which is commonly positive in the sarcomatous component of carcinosarcoma, were not performed in this case, the overall morphological and immunohistochemical profile (negative for epithelial markers AE1/AE3 and CAM5.2, and focally positive for muscle markers such as α-SMA, desmin, and caldesmon) strongly supported the diagnosis of a sarcomatous component. These findings are consistent with previously reported carcinosarcomas.^15)^ Carcinoma cells in this case proliferated within the mucosal epithelium and infiltrated the stroma beneath, creating tiny cellular nests. We reviewed the case after the esophagectomy and observed the biopsy sample from the original esophageal squamous cell carcinoma. Esophageal squamous cell carcinoma with poor differentiation was noted, suggesting the presence of sarcomatous components, and the diagnosis was revised to indicate a high probability of carcinosarcoma. The initial esophageal lesion was a 0-IIb superficially flat lesion, distinct from the typical polypoid growth, which is not a typical endoscopic finding of esophageal carcinosarcoma. This is the major reason why esophageal carcinosarcoma was initially diagnosed as esophageal squamous cell carcinoma. There have also been several reports of the simultaneous presentation of esophageal squamous cell carcinoma and esophageal leiomyosarcoma,^16)^ and the possibility that squamous cell carcinoma may affect the growth of leiomyosarcoma via cytokines and growth factors.^17)^ In such cases, squamous cell carcinoma is thought to be present in the form of microinvasions in the smooth muscle tumor mass.^18)^ However, the present case does not appear to fit this description. The current case occurred at 9 months after irradiation, which is the shortest incubation period ever reported for a radiation-induced sarcoma. Given that the average latency period of radiation-induced sarcoma is reported to be 14.1 years,^18)^ the latency in the present case was extremely short. Radiation-associated carcinosarcomas of the esophagus are extremely rare, although several cases have been reported.^19)^ The latency period in those reports ranged from several years to several decades, making radiation induction unlikely. Histopathological findings of atypical spindle-shaped and round cells irregularly distributed within the esophageal wall may suggest an increased likelihood that the carcinomatous component of esophageal carcinosarcoma could be eliminated by radiotherapy. This case demonstrates that the sarcomatous component of esophageal carcinosarcoma may persist even when the carcinomatous component shows a complete response to chemoradiotherapy. Recognizing this differential response is crucial, as it emphasizes the need for vigilant endoscopic surveillance and timely consideration of salvage resection.

Moreover, this case provides valuable insight into the treatment strategy for this rare tumor and may contribute to improved decision-making in similar cases in the future.

## CONCLUSIONS

In this rare case of esophageal carcinosarcoma, dCRT resulted in the complete disappearance of the carcinomatous component, while the sarcomatous component persisted and required surgical resection. This finding indicates that the two components of carcinosarcoma may exhibit markedly different sensitivities to chemoradiotherapy. Recognizing this characteristic is essential for determining appropriate treatment strategies in patients with this uncommon tumor. To the best of our knowledge, no similar cases have been reported.

## DECLARATIONS

### Availability of data and materials

The datasets supporting the conclusions of this study are available from the corresponding author.
